# Comparing the MSIS-29 and the Health Utilities Index Mark III in Multiple Sclerosis

**DOI:** 10.3389/fneur.2021.747853

**Published:** 2021-12-17

**Authors:** Ruth Ann Marrie, Casandra Dolovich, Gary R. Cutter, Robert J. Fox, Amber Salter

**Affiliations:** ^1^Department of Internal Medicine, Max Rady College of Medicine, Rady Faculty of Health Sciences, University of Manitoba, Winnipeg, MB, Canada; ^2^Department of Community Health Sciences, Max Rady College of Medicine, Rady Faculty of Health Sciences, University of Manitoba, Winnipeg, MB, Canada; ^3^Department of Biostatistics, University of Alabama at Birmingham, Birmingham, AL, United States; ^4^Mellen Center for Multiple Sclerosis, Neurological Institute, Cleveland Clinic, Cleveland, OH, United States; ^5^Department of Neurology, UT Southwestern University, Dallas, TX, United States

**Keywords:** Multiple Sclerosis, validity, reliability, quality of life, discriminating ability

## Abstract

**Objective:** Since the properties of health-related quality of life measures vary across samples, studies directly comparing the properties of different measures can be useful in understanding their relative strengths and limitations. We aimed to compare the psychometric properties of the Health Utilities Index Mark III (HUI3) and the Multiple Sclerosis Impact Scale-29 (MSIS-29).

**Methods:** In Spring 2020, North American Research Committee on Multiple Sclerosis (NARCOMS) Registry participants completed the HUI3, MSIS-29, Patient Determined Disease Steps (PDDS) and SymptoMScreen. For the HUI3 and MSIS-29 we assessed floor and ceiling effects, construct validity, and internal consistency reliability. We used relative efficiency to compare the discriminating ability of the two measures with respect to disability.

**Results:** We included 5,664 participants in the analysis, with mean (SD) age 63 (10.1) years; 4,579 (80.8%) were women. For the HUI3 the mean (SD) score was 0.44 (0.32), for the MSIS-29 physical it was 34.0 (24.2) and for the MSIS-29 psychological it was 25.9 (20.4). Neither of the measures had floor or ceiling effects, and internal consistency reliability was > 0.70 for both. The HUI3 and MSIS-29 physical were strongly correlated (r = −0.78; 95%CI:−0.79,−0.77). The correlation between the HUI3 and MSIS-29 psychological was weaker but remained moderately strong (r = −0.64; 95%CI:−0.66,−0.63). After adjusting for sociodemographic and clinical factors, relative efficiency to discriminate between disability (PDDS) groups was highest for the MSIS-29 physical scale, followed by the HUI3.

**Conclusion:** Both measures had adequate validity and reliability. The MSIS-29 physical discriminated between disability groups better than the HUI3.

## Introduction

Multiple Sclerosis (MS) is associated with reduced health-related quality of life (HRQOL) (1), a key patient-reported outcome in observational studies and clinical trials (2). Quality of life is a person's sense of well-being or satisfaction with important areas of life. HRQoL is a more discrete construct and refers to the value one places on abilities and limitations, including the effects of disease and its treatments upon physical, emotional and social well-being (3). Studies of HRQOL in MS populations have used generic and disease-specific measures (1, 4). Generic measures have the advantage of relevance to people in various health states and comparability between different disease states (3). For example, using the Short Form-36 (SF-36) people with MS reported lower HRQOL than the general population (5), or people with epilepsy and diabetes (6). Similarly, people with MS reported lower HRQOL than persons with rheumatoid arthritis or spinal cord injury using the Disability and Impact Profile (7). However, generic measures may not capture all aspects of HRQOL relevant to a particular disease, for which disease-specific measures may be superior.

Previous studies of HRQOL used multiple generic and disease-specific measures in studies of MS populations (1, 4). The choice of measure should depend on the purpose for which the measure is being used, such as in clinical practice or in a clinical trial, as well as the psychometric properties of the instrument, such as validity and reliability. Measures with good psychometric properties provide more accurate assessments of HRQOL than those with weak properties. A prior review of generic preference-based (utility) measures in MS including the EuroQol five-dimensional questionnaire, the Short Form-6D, the Assessment of Quality of Life, the Quality of Well-Being Scale and the Health Utilities Index Mark III (HUI3), found that the HUI3 had the strongest psychometric properties (8). In the North American Research Committee on Multiple Sclerosis (NARCOMS) population we found that the HUI3 was more responsive to changes in disability than the RAND-12 (9).

The Multiple Sclerosis Impact Scale-29 (MSIS-29) was developed in 2001. Unlike other commonly used disease-specific measures of HRQOL, it was not developed by adding MS-specific measures to an existing generic measure. In contrast, the MSIS-29 was developed by generating a pool of potential questionnaire items through literature review, expert opinion, and interviews of people with MS, followed item reduction, development and testing of the instrument (10). In one study, the MSIS-29 performed well as compared to the generic Short Form-36, and the disease-specific Functional Assessment of Multiple Sclerosis (11). Since the properties of HRQOL measures vary across samples, studies directly comparing the properties of different measures can be useful in understanding their relative strengths and limitations. Therefore, we aimed to directly compare the psychometric properties of the HUI3 to the MSIS-29.

## Methods

### Study Population

Study participants were drawn from the North American Research Committee on Multiple Sclerosis (NARCOMS) Registry. As described elsewhere, the NARCOMS Registry is a self-report registry for persons with MS. Participants report demographic and clinical information at enrollment and semi-annually (“updates”) thereafter either via paper questionnaires or online. Prior work has shown the validity of self-reported diagnoses of MS in the NARCOMS Registry and of the disability measures used (12, 13). Participants agree to the use of their de-identified information for research. The NARCOMS registry and its surveys are approved by the IRB at Washington University in St. Louis.

### Demographic and Clinical Characteristics

At the time of enrollment, participants report gender, date of birth, the highest level of education attained, race, ages at MS symptom onset and diagnosis. Education level was categorized as high school/GED or less, Associate's Degree or Technical Degree, Bachelor's Degree or Post-graduate education. Race was categorized as white vs. non-white. Disease duration was calculated based on age of MS symptom onset.

Annual household income was reported in the Spring 2020 update as < $15,000; $15000–$30,000; $30,001-$50,000; $50,000-$100,000 or >$100,000. Disability status was reported using Patient Determined Disease Steps (PDDS) which correlates highly with a clinician-scored Expanded Disability Status Scale score. Consistent with our prior work evaluating the HUI3, we grouped PDDS 0–1 as mild disability (no limitations in mobility), 2–4 as moderate disability (moderate limitations in mobility), and 5–8 as severe disability (significant limitations in mobility) (14). Participants completed the SymptoMScreen which assesses symptoms related to MS in the domains of walking/mobility, hand function/dexterity, spasticity/stiffness, bodily pain, sensory symptoms, bladder control, fatigue, vision, dizziness, cognitive function, depression and anxiety (15, 16). Each domain is assessed using a single item with 7 options ranging from 0 (not affected at all) to 6 (total limitation). A composite score (SMSS) can be calculated as a sum of responses across all domains, with values ranging from 0 to 84; higher scores indicate more botheration. Participants also reported the use of disease-modifying therapy in the prior 6 months, which we classified as any vs. none.

### Health-Related Quality of Life

In the Spring 2020 update survey, we measured HRQOL using the HUI3 and the MSIS-29. The HUI3 is a generic measure of health utility comprised of 15 items with demonstrated validity and reliability in MS populations (17). These items assess health state with respect to eight attributes: vision, hearing, speech, mobility, dexterity, emotion, cognition and pain (18). The single attribute scores can be summarized into a multi-attribute score for which the values range from 0 (death) to 1 (perfect health). Negative values (as low as−0.36) are permitted, and constitute negative health states that are considered worse than death (18). The MSIS-29 is a disease-specific measure of HRQOL comprised of 29 items, 20 addressing the physical impact of MS and 9 addressing the psychological impact of MS (10). Each item has five response items from 1 (not at all) to 5 (extremely). The responses are summed across items to create physical and psychological scales. The scores are then transformed to range from 0 to 100; 100 indicates worse health. Due to an error, responses to item 16 were not captured from online respondents. If a respondent misses an item but has at least half of the items in the scale completed a score can still be determined (10). The approach used is as follows: the completed items in the scale are summed, divided by the number of items completed, and the preceding mean score is used as the score for each of the missing items, following which the usual scoring procedure can be used.

### Analysis

We included participants who responded to the Spring 2020 survey, reported a physician-confirmed diagnosed of MS, had complete information regarding age and gender, and who fully completed the HUI3 because the HUI3 is intolerant to missing data and does not provide rules for imputing missing data. We summarized characteristics of the respondents using means [standard deviation (SD)], median [interquartile range (IQR)], and frequency (percent) as appropriate.

For each HRQOL measure we determined the mean (SD), skewness of the distribution of scores, and the percentage of participants scoring the minimum (floor) and maximum (ceiling) possible scores. Ideally, mean scores are near the scale's mid-point, floor and ceiling effects are <15%, and skewness statistics range from −1 to 1. There is no gold standard for HRQOL but we calculated the Pearson correlations [95% confidence intervals (95%CI)] between the HRQOL measures to evaluate concurrent validity. We also assessed construct validity (through hypothesis testing), and internal consistency reliability (19). We expected high composite scores for the SymptomMScreen to be associated with worse HRQOL; so we assessed these associations using Spearman correlations (95%CI). We assessed the ability of each HRQOL measure to distinguish between disability groups (discriminative validity) using one-way analysis of covariance (ANCOVA), adjusting for age, gender, race, education, income, disease duration and use of disease-modifying therapy. Disability group (as defined above) was included as the intergroup factor, and the HRQOL measure was the dependent variable. We calculated the relative efficiency (RE) of the HRQOL measure to discriminate between groups as the ratio of the between group ANCOVA F-statistics. The measure with the largest F-statistic is selected as the reference category to which the other measure is compared. We assessed the internal consistency of each screening tool using Cronbach's alpha (α), where an acceptable α was ≥0.70 (20).

Statistical analyses used SAS V9.4 (SAS Institute Inc., Cary, NC).

## Results

### Participants

The Spring 2020 survey was distributed to 10,202 NARCOMS participants, of whom 6,385 (62.6%) responded. As compared to responders, non-responders were more likely to be African American, male, had one year less education, and were two years younger. Most of these differences were statistically significant but small in magnitude (Supplementary Table e1). After excluding individuals who did not report a physician-confirmed diagnosis of MS (*n* = 198), with missing date of birth (*n* = 244), gender (*n* = 1), and one or more items on the HUI (*n* = 278), the final sample included 5,664 participants. Most participants were white, with more than a high school education, and moderate or severe disability based on the PDDS (Table 1).

**Table 1:** Participant characteristics.

**Characteristics**	**All Responders**	**Online Responders**	**Paper Responders**	**P-value^1^**
	**(*N* = 5,664)**	**(*N* = 4,183)**	**(*N* = 1,481)**	
Age at time of Spring 2020 survey (years)^a^, mean (SD)	63 (10.1)	62 (9.9)	67 (9.3)	**<0.0001**
Age at MS symptom onset (years)^b^, mean (SD)	31 (10.3)	31 (10.3)	30 (10.6)	**0.0078**
Age at MS diagnosis (years)^c^, mean (SD)	39 (9.9)	39 (9.8)	39 (10.2)	0.79
Female^d^, *n* (%)	4,579 (80.8)	3,337 (79.8)	1,242 (83.9)	**0.0005**
**Race**^**e**^, ***n*** **(%)**				0.37
White	4,933 (87.1)	3,653 (87.3)	1,280 (86.4)	
Non-white	730 (12.9)	529 (12.7)	201 (13.6)	
**Education** ^**f**^ **, n (%)**				**<0.0001**
High school/GED or less	1,448 (26.7)	879 (22.1)	569 (39.7)	
Associate's/Technical degree	924 (17.1)	657 (16.5)	267 (18.6)	
Bachelor's degree or Post-grad	3,048 (56.2)	2,450 (61.5)	598 (41.7)	
**Annual household income** ^**g**^ **, n (%)**				**<0.0001**
< $15,000	318 (5.7)	161 (3.9)	157 (11.0	
$15,001–30,000	740 (13.3)	460 (11.1)	280 (19.6)	
$30,001–50,000	785 (14.1)	564 (13.6)	221 (15.5)	
$50,001–100,000	1,399 (25.1)	1,120 (27.02)	279 (19.5)	
Over $100,000	1,112 (19.9)	974 (23.5)	138 (9.7)	
I do not wish to answer	1,219 (21.9)	866 (20.89)	353 (24.7)	
PDDS^h^, median (p25–p75)	4 (1–6)	3 (1–6)	4 (2–6)	**<0.0001**
**PDDS**				**<0.0001**
Mild (0–1)	1,641 (29.4)	1,327 (32.1)	314 (21.6)	
Moderate (2–4)	1,833 (32.8)	1,360 (32.9)	473 (32.5)	
Severe (5–8)	2,113 (37.8)	1,445 (35.0)	668 (45.9)	
SymptoMScreen, median (p25–p75)	20 (12–19)	19 (11–28)	23 (14–33)	**<0.0001**
Disease Duration (years)^i^, mean (SD)	32.11 (12.1)	30.53 (11.7)	36.66 (12.4)	**<0.0001**
**Disease-modifying therapy**, ***n*** **(%)**				**<0.0001**
Any	2,885 (50.9)	2,277 (54.4)	608 (41.1)	
No	2,779 (49.1)	1,906 (45.6)	873 (58.9)	

*a, 0 missing; b, 71 missing; c, 30 missing; d, 0 missing; e, 1 missing; f, 244 missing; g, 91 missing; h, 77 missing; i, 73 missing*.

1 *T-test for continuous variables; Fisher's exact test for categorical variables aside from chi-square test for annual household income; non-parametric Wilcoxon signed rank test for continuous PDDS and SymptoMScreen. Bold indicates statistical significance*.

### Health-Related Quality of Life

The mean (SD) HUI3 score was 0.44 (0.32). For the MSIS-29 physical the mean (SD) score was 34.0 (24.2) whereas it was 25.9 (20.4) for the MSIS-29 psychological. On the HUI3, 57 (1.01%) participants scored the maximum value of 1 and none scored the minimum value; the lowest value scored was −0.34 (*n* = 1). On the MSIS-29 physical, 3.8% participants scored the maximum value of 100 and 0.37% participants scored the minimum value. On the MSIS-29 psychological, 7.5% participants scored the maximum value and 0.35% scored the minimum value. Internal consistency reliability, as measured by Cronbach's alpha, was acceptable for both measures, but higher for the MSIS-29 than the HUI3 as demonstrated by the non-overlapping confidence intervals (Table 2).

**Table 2:** Acceptability and reliability of the measures of health-related quality of life.

	**HUI32**	**MSIS-29**	
		**Physical**	**Psychological**
Items	15	20	9
Levels	5	5	5
Mean (SD)	0.44 (0.32)	34.0 (24.2)	25.9 (20.4)
Median (IQR)	0.44 (0.19–0.70)	30.3 (13.8–51.3)	22.2 (11.1–38.9)
Skewness, Kurtosis	(−0.124,−0.896)	(0.523,−0.586)	(0.943,0.594)
Best score	1	0	0
Worst score	−0.343	100	100
Floor (%)	0.02%	0.37%	0.35%
Ceiling (%)	1.01%	3.85%	7.51%
**Cronbach's alpha (95% CI)**
Overall	0.808 (0.80,0.82)	0.962 (0.95,0.97)^*^	0.914 (0.90,0.93)
Online	0.802 (0.79,0.82)	0.962 (0.95,0.97)	0.908 (0.89,0.93)
Paper	0.812 (0.79,0.84)	0.961 (0.94,0.98)	0.923 (0.89,0.95)
SymptoMScreen score (ρ, 95% CI)	−0.799 (−0.81,−0.79)	0.838 (0.83,0.85)	0.717 (0.70,0.73)
Age 2020 (ρ, 95% CI)	−0.186 (−0.21,−0.16)	0.199 (0.20,0.22)	−0.030 (−0.03,0)

* *Calculated without the missing question (item #16)*.

The HUI3 and MSIS-29 physical were strongly correlated (r = −0.78; 95%CI:−0.79,−0.77). The correlation between the HUI3 and MSIS-29 psychological was weaker but remained moderately strong (r = −0.64; 95%CI:−0.66,−0.63). As expected, SymptoMScreen was strongly correlated with the HUI3 and the MSIS-29 physical and psychological scores, but age was weakly correlated with HUI3 and the MSIS-29 physical and psychological scores (Table 2).

When we examined relative efficiency of the HRQOL measures to distinguish between disability groups overall after accounting for covariates, relative efficiency was highest for the MSIS-29 physical scale, followed by the HUI3 (Table 3). The HUI3 had higher relative efficiency than the MSIS-29 when discriminating between mild vs. moderate disability, but lower relative efficiency when discriminating between mild vs. severe, or moderate vs. severe disability. These findings were similar when we stratified by online vs. paper responses (Supplementary Table e2).

**Table 3:** Analysis of covariance for health-related quality of life measures by disability level^*^.

	**HUI32**		**MSIS-29**			
			**Physical**		**Psychological**	
**Independent factor variable**	***F*-test**	***p*-value**	***F*-test**	***p*-value**	***F*-test**	***p*-value**
Disability status	1331.6	**<0.0001**	1893.5	**<0.0001**	222.65	**<0.0001**
*Relative efficiency*	0.70		1.0		0.12	
Mild vs. Moderate	1025.59	**<0.0001**	883.6	**<0.0001**	321.41	**<0.0001**
*Relative efficiency*	1.0		0.86		0.31	
Mild vs. Severe	2652.82	**<0.0001**	3741.28	**<0.0001**	377.47	**<0.0001**
*Relative efficiency*	0.71		1.0		0.10	
Moderate vs. Severe	492.45	**<0.0001**	1180.56	**<0.0001**	2.96	0.0852
*Relative efficiency*	0.42		1.0		0.003	

* *Adjusted for age (continuous), gender, race, education and income. Bold indicates statistical significance*.

## Discussion

Appropriate selection of study measures depends on an understanding of how they will perform in the population of interest. In this large cross-sectional study involving over 5,000 participants with MS we compared the psychometric properties of the HUI3 to the MSIS-29. With respect to item content, the HUI3 has items that assess vision, hearing, speaking, pain, mobility, dexterity, cognition, mood, and ability to perform independent activities of daily living such as bathing. It does not capture fatigue. The MSIS-29 does not assess vision, hearing, speaking or pain, but does capture mental fatigue, items related to hand function, mobility/balance, motor symptoms such as tremor and spasms, mood, bladder urgency, and need for assistance with activities. We found that neither of the measures had significant floor or ceiling effects, and both had mean values near the midpoint of the scales. Internal consistency reliability was acceptable for both measures but was higher for the MSIS-29 physical than for the HUI3, which in turn had better reliability than the MSIS-29 psychological. Based on correlations with the SymptoMScreen the HUI3, MSIS-29 physical and psychological scales demonstrated construct validity. Although both discriminated between disability groups, as assessed by the PDDS, the MSIS-29 physical scale had greater discriminating ability overall, and for discriminating between participants with mild vs. severe, or moderate vs. severe disability. The HUI3 had greater ability to discriminate between participants with mild vs. moderate disability. The generally superior discriminating ability of the MSIS-29 physical may reflect the fact that it is a disease-specific measure which incorporated feedback from people MS during the development phase whereas the HUI is generic.

We were not able to identify any other studies that have compared the performance of the HUI3 and the MSIS-29, but there has been other work comparing the HUI3 and MSIS-29 to other measures. Prior cross-sectional studies have found that the HUI3 is superior to the Physical Component Score of the SF-36 for discriminating between individuals with MS who have moderate and severe disability (17, 21), and that the HUI3 is more responsive than the RAND-12 to changes in disability or employment status. Notably, the Instrumental Activities of Daily living (IADL) scale is also better at discriminating between individuals with moderate or severe disability than the RAND-12 (22). It captures dependence for some activities, as does the MSIS-29 (e.g., having to depend on others, problems using transport). A study of 121 hospitalized people with MS compared the properties of the MSIS-29, SF-36 and Functional Assessment of Multiple Sclerosis. In that study, the MSIS-29 physical was the most responsive physical scale whereas the FAMS emotional was the most responsive psychosocial scale (23). In another study involving three hospital samples and a community sample who completed the SF-36 and MSIS-29, the SF-36 had floor effects (>31% of respondents had the minimum score) but the MSIS-29 physical did not show floor or ceiling effects.

Our findings should be considered in light of study limitations. The survey response rate was 62.6%. While lower than desirable this is consistent with response rates for medical surveys which average about 60%. Responders differed from non-responders. Participants in the NARCOMS registry voluntarily enroll, leading to potential selection bias. However, the mean age of study participants is comparable to the peak age-specific prevalence of MS in the United States, and prior findings in the NARCOMS population have been replicated in external populations. For online respondents responses to item 16 were not captured which could have influenced our findings, but our complementary analyses stratified by online vs. paper response and the conclusions were similar. We compared several properties of the HUI3 and MSIS-29 but did not consider all relevant properties such as responsiveness. We assessed the ability of the two measures to discriminate between groups with differing disability levels defined based on ambulation, but did not assess the ability to discriminate between groups differing with respect to other relevant factors such as cognition, upper limb function or vision. These issues could be addressed in future studies.

## Conclusion

The HUI3 and MSIS-29 showed adequate validity and reliability in our study population. Based on relative efficiency, the MSIS-29 showed greater ability to discriminate between disability groups than the HUI3.

## Data Availability Statement

The datasets presented in this article are not readily available because the datasets generated and analyzed for this study are held by the NARCOMS Registry (https://www.narcoms.org). Requests to access the datasets should be directed to MSregistry@narcoms.org.

## Ethics Statement

The studies involving human participants were reviewed and approved by Washington University at St. Louis Institutional Review Board. Written informed consent for participation was not required for this study in accordance with the national legislation and the institutional requirements.

## Author Contributions

RM and AS conceived of the idea. CD conducted the statistical analyses. RM drafted the manuscript. RM, CD, GC, RF, and AS revised the manuscript and approved of the final version. All authors contributed to the article and approved the submitted version.

## Funding

NARCOMS is a project of the Consortium of Multiple Sclerosis Centers (CMSC). NARCOMS is funded in part by the CMSC and the Foundation of the CMSC. The study was also supported in part by the Waugh Family Chair in Multiple Sclerosis and Research Manitoba Chair (to RM). The funding source(s) had no role in the study design, collection, analysis or interpretation of the data, nor in the decision to submit the article for publication.

## Conflict of Interest

RM receives research funding from: CIHR, Research Manitoba, Multiple Sclerosis Society of Canada, Multiple Sclerosis Scientific Foundation, Crohn's and Colitis Canada, National Multiple Sclerosis Society, CMSC and the US Department of Defense, and is a co-investigator on studies receiving funding from Biogen Idec and Roche Canada. GC data/safety monitoring committees for AMO, BioLineRx, BrainStorm Cell Therapeutics, Galmed, Horizon, Hisun, Merck, Merck/Pfizer, OPKO Biologics, Neurim, Novartis, Orphazyme, Sanofi, Reata, Receptos/Celgene, Teva, NHLBI (Protocol Review Committee), NICHD (OPRU oversight committee); consulting/advisory boards for Biogen, Click Therapeutics, Genzyme, Genentech, GW, Klein Buendel, MedImmune, MedDay, Novartis, Osmotica, Perception Neuroscience, Recursion, Roche, Somahlution, and TG Therapeutics. RF has received personal consulting fees from AB Science, Biogen, Celgene, EMD Serono, Genentech, Genzyme, Immunic, Janssen, Novartis, Sanofi, and TG Therapeutics; has served on advisory committees for AB Science, Biogen, Genzyme, Immunic, Janssen, Novartis, Sanofi, and TG Therapeutics; and received clinical trial contract and research grant funding from Biogen, Novartis, and Sanofi. AS is a journal editor/member of editorial advisory board for Circulation: Cardiovascular Imaging. The remaining author declares that the research was conducted in the absence of any commercial or financial relationships that could be construed as a potential conflict of interest.

## Publisher's Note

All claims expressed in this article are solely those of the authors and do not necessarily represent those of their affiliated organizations, or those of the publisher, the editors and the reviewers. Any product that may be evaluated in this article, or claim that may be made by its manufacturer, is not guaranteed or endorsed by the publisher.
