# Supplementary material for: Comparing the MSIS-29 and the Health Utilities Index Mark III in Multiple Sclerosis
Source: Front Neurol. 2021 Dec 17;12:747853. doi: 10.3389/fneur.2021.747853 (PMC8718449; doi:10.3389/fneur.2021.747853)
Supplement: Supplementary file 1 [file Table_1.DOCX]

Table e1. Characteristics of responders and non-responders

|  |  |  |  |
| --- | --- | --- | --- |
| **Characteristic** | **Non-responders (N=3817)** | **Responders (N=6385)** | **P-value** |
| Race, n (%) |  |  | **<0.001^c^** |
| Black/AA | 146 (3.8) | 146 (2.3) |  |
| Other | 520 (13.6) | 706 (11.1) |  |
| White | 3151 (82.6) | 5533 (86.7) |  |
| Gender*, n (%) |  |  | **0.009^c^** |
| Women | 2997 (78.7) | 5157 (80.9) |  |
| Men | 810 (21.3) | 1221 (19.1) |  |
| Age in 2020*, median (IQR) | 59.0 [49.0,66.0] | 62.0 [55.0,68.0] | **<0.001^b^** |
| Number of Years of Education*, median (IQR) | 15.0 [13.0,17.0] | 16.0 [14.0,18.0] | **<0.001^b^** |
| Age of Symptom Onset (yrs)*, median (IQR) | 30.0[23.0,38.0] | 30.0[24.0,38.0] | **0.012^b^** |
| Age at diagnosis (yrs)*, median (IQR) | 38.0[31.0,46.0] | 39.0[31.0,46.0] | 0.22^b^ |
| Age at Enrollment*, median (IQR) | 48.0[40.0,55.0] | 48.0[41.0,54.0] | 0.078^b^ |
| PDDS at Enrollment*, median (IQR) | 3.0[1.00,4.0] | 3.0[1.00,4.0] | **<0.001^b^** |
| *Data not available for all participants. Missing values: Sex = 17, Age in 2020 = 624, Level of Education = 1113, Number of Years of Education = 7254, Age Symptom Onset = 954, Age at diagnosis = 837, Age at Enrollment = 626, PDDS at Enrollment = 1291, p-values: a=ANOVA, b=Kruskal-Wallis test, c=Pearson's chi-square test, d=Fisher's Exact test. | | | |

Table e2A. Analysis of covariance for health-related quality of life measures by disability level for online respondents*

|  | **HUI32** | | **MSIS-29** | | | |
| --- | --- | --- | --- | --- | --- | --- |
|  |  |  | **Physical** | | **Psychological** | |
| **Independent Factor Variable** | **F-test** | **p-value** | **F-test** | **p-value** | **F-test** | **p-value** |
| Disability Status | 1051.09 | **<0.0001** | 1536.23 | **<0.0001** | 165.25 | **<0.0001** |
| *Relative efficiency* | 0.68 |  | 1.0 |  |  |  |
| Mild vs. Moderate | 777.99 | **<0.0001** | 640.82 | **<0.0001** | 233 | **<0.0001** |
| *Relative efficiency* | 1.0 |  | 0.82 |  |  |  |
| Mild vs. Severe | 2088.95 | **<0.0001** | 3036.55 | **<0.0001** | 276.96 | **<0.0001** |
| *Relative efficiency* | 0.69 |  | 1.0 |  |  |  |
| Moderate vs. Severe | 394.19 | **<0.0001** | 999.5 | **<0.0001** | 2.38 | 0.12 |
| *Relative efficiency* | 0.39 |  | 1.0 |  |  |  |

*Adjusted for age (continuous), gender, race, education and income; **BOLD** indicates p<0.05

Table e2B. Analysis of covariance for health-related quality of life measures by disability level for paper respondents*

|  | **HUI32** | | **MSIS-29** | | | |
| --- | --- | --- | --- | --- | --- | --- |
|  |  |  | **Physical** | | **Psychological** | |
| **Independent Factor Variable** | **F-test** | **p-value** | **F-test** | **p-value** | **F-test** | **p-value** |
| Disability Status | 354.66 | **<0.0001** | 449.05 | **<0.0001** | 70.56 | **<0.0001** |
| *Relative efficiency* | 0.79 |  | 1.0 |  | 0.16 |  |
| Mild vs. Moderate | 279.65 | **<0.0001** | 246.69 | **<0.0001** | 101.89 | **<0.0001** |
| *Relative efficiency* | 1.0 |  | 0.88 |  | 0.36 |  |
| Mild vs. Severe | 709.23 | **<0.0001** | 881.65 | **<0.0001** | 126.54 | **<0.0001** |
| *Relative efficiency* | 0.80 |  | 1.0 |  | 0.14 |  |
| Moderate vs. Severe | 123.23 | **<0.0001** | 247.76 | **<0.0001** | 1.13 | 0.29 |
| *Relative efficiency* | 0.50 |  | 1.0 |  | 0.005 |  |

*Adjusted for age (continuous), gender, race, education and income; **BOLD** indicates p<0.05
